# Plasma Markers of Neurodegeneration Are Raised in Friedreich’s Ataxia

**DOI:** 10.3389/fncel.2018.00366

**Published:** 2018-10-30

**Authors:** Anna M. Zeitlberger, Gilbert Thomas-Black, Hector Garcia-Moreno, Martha Foiani, Amanda J. Heslegrave, Henrik Zetterberg, Paola Giunti

**Affiliations:** ^1^Ataxia Centre, Department of Clinical and Movement Neurosciences, UCL Queen Square Institute of Neurology, University College London, London, United Kingdom; ^2^National Hospital for Neurology and Neurosurgery, University College London Hospitals Foundation NHS Trust, London, United Kingdom; ^3^UK Dementia Research Institute, University College London, London, United Kingdom; ^4^Department of Neurodegenerative Disease, UCL Queen Square Institute of Neurology, University College London, London, United Kingdom; ^5^Clinical Neurochemistry Laboratory, Sahlgrenska University Hospital, Mölndal, Sweden; ^6^Department of Psychiatry and Neurochemistry, Institute of Neuroscience and Physiology, Sahlgrenska Academy, University of Gothenburg, Mölndal, Sweden

**Keywords:** biomarkers, Friedreich’s ataxia, neurofilament-light chain, glial fibrillary acidic protein, tau, ubiquitin C-terminal hydrolase L1

## Abstract

**Background:** Friedreich’s ataxia (FRDA) is the most common autosomal recessive ataxia. Disease-modifying treatments are not available yet; however, several compounds are currently under investigation. As a result, there is a growing need for the identification of robust and easily accessible biomarkers for the monitoring of disease activity and therapeutic efficacy. The simultaneous measurement of multiple brain-derived proteins could represent a time- and cost-efficient approach for biomarker investigation in pathologically complex neurodegenerative diseases like FRDA.

**Objectives:** To investigate the role of plasma neurofilament-light chain (NfL), glial fibrillary acidic protein (GFAP), total tau (t-tau) and ubiquitin C-terminal hydrolase L1(UCHL1) as biomarkers in FRDA. Additionally, NfL measurements derived from the novel multiplex assay were compared to those from an established NfL singleplex assay.

**Methods:** In this study, an ultrasensitive Single molecule array (Simoa) 4-plex assay was used for the measurement of plasma NfL, GFAP, t-tau, and UCHL1 in 33 FRDA patients and 13 age-matched controls. Differences in biomarker concentrations between these groups were computed and associations with genetic and disease related parameters investigated. Additionally, the agreement between NfL measurements derived from the 4-Plex and an established Simoa NfL singleplex assay was assessed.

**Results:** Mean plasma NfL, GFAP and UCHL1 levels were significantly higher in FRDA patients than in controls (NfL: *p <* 0.001; GFAP: *p* = 0.006, and UCHL1: *p* = 0.020). Conversely, there was no significant difference in concentrations of t-tau in the patient and control group (*p* = 0.236). None of the proteins correlated with the GAA repeat length or the employed measures of disease severity. The individual NfL values derived from the two assays showed a strong concordance (*r_c_* = 0.93). Although the mean difference of 1.29 pg/mL differed significantly from 0 (*p* = 0.006), regression analysis did not indicate the presence of a proportional bias.

**Conclusion:** This is the first study demonstrating that NfL, GFAP, and UCHL1 levels are raised in FRDA, potentially reflecting ongoing neuronal degeneration and glial activation. Further studies are required to determine their role as marker for disease activity and progression. Furthermore, the novel 4-plex assay appears to be a valid tool to simultaneously measure brain-derived proteins at extremely low concentrations in the peripheral circulation.

## Introduction

Friedreich’s ataxia (FRDA) is the most common autosomal recessive ataxia worldwide, affecting 1 in 20,000 Caucasians (Ruano et al., 2014). In the majority of patients, it is caused by a homozygous guanine-adenine-adenine (GAA) expansion located in the first intron of the *frataxin* (*FXN*) gene (Campuzano et al., 1996). This leads to a deficiency of the mitochondrial protein FXN that in turn causes degeneration in the dorsal root ganglia, spinocerebellar, and corticospinal tracts and the cerebellar dentate nucleus (Koeppen et al., 2017). This pattern ultimately gives rise to the complex clinical phenotype characterized by a progressive mixed sensory and cerebellar ataxia with depressed reflexes, limb weakness, and impaired proprioception (Parkinson et al., 2013). The clinical presentation is often complicated by the high incidence of skeletal deformities, insulin resistance, and cardiac dysfunction (Delatycki and Corben, 2012). In addition to the damage in neurons, cardiac myocytes, and pancreatic beta-cells, there is a growing number of evidence indicating that *FXN* mutations also cause astrocyte impairment (Loriìa and Diìaz-Nido, 2015; Franco et al., 2017).

**FIGURE 1:**
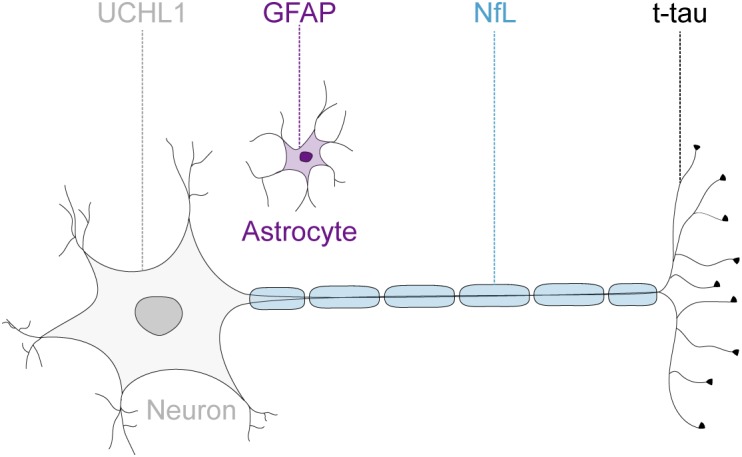
Neuronal and glial biofluid markers of neurodegeneration. The glial fibrillary acidic protein (GFAP) is the main structural protein in astrocytes. The neurofilament-light chain protein (NfL) reflects damage to large-caliber myelinated axons. The microtubule binding protein tau is preferentially localized within thin, non-myelinated axons. The ubiquitin C-terminal hydrolase L1 (UCHL1) is one of the most abundant proteins in the central nervous system and localized mainly in neuronal cytoplasm.

Given the advances in the understanding of the pathogenesis of FRDA (Cook and Giunti, 2017), and the consequently rapidly expanding therapeutic pipeline (Rummey et al., 2018), there is an increasing need to measure disease progression and monitor drug effects reliably and objectively. Previous trials have been mainly reliant on clinical measurements, which are susceptible to inter-rater variability and limited by their poor ability to detect disease progression in this slowly progressive disease (Bürk et al., 2009). The Food and Drug Administration defines biomarkers as objectively measurable characteristics that are indicators of physiological and pathological processes or reflect response to therapeutic interventions (Biomarkers Definitions Working Group, 2001). Over the past decade, several brain-derived proteins have emerged as promising candidate markers for neurodegeneration in a variety of acute and chronic neurological diseases (Zetterberg et al., 2013; Burman et al., 2014; Benninger et al., 2016; Byrne et al., 2017). These include the astrocytic intermediate filament protein, glial fibrillary acidic protein (GFAP) (Yang and Wang, 2015); the neuron-specific cytoskeletal protein neurofilament-light chain (NfL) (Liu et al., 2004); a cytoplasmatic neuronal enzyme, ubiquitin C-terminal hydrolase L1 (UCHL1) (Wilkinson et al., 1989); and the microtubule associated protein tau (Binder et al., 1985). While NfL, total tau (t-tau) and UCHL1 are recognized as markers for neuroaxonal damage (Kawata et al., 2016), increased GFAP levels reflect astrocyte activation or injury (Yang and Wang, 2015; Figure 1). These proteins are released into the extracellular space after neuronal or glial injury and subsequently detectable in the cerebrospinal fluid (CSF). The development of ultrasensitive immunoassays now allows the quantification of these proteins at extremely low abundance in the peripheral circulation (Rissin et al., 2010). Moreover, the introduction of multi-analyte, or multiplex, assays enables the simultaneously quantification of multiple proteins within one experiment. However, multiplex assay have not been adopted widely as concerns remain regarding the potential interference between different antibodies, analytes or assay reagents (Tighe et al., 2015).

Considering that the nervous system is the predominantly affected tissue in FRDA, the investigation of brain-enriched proteins reflecting damage to different central nervous system cell types appeared as a promising approach of biomarker research in this disease. In this study, we aimed to investigate whether plasma GFAP, NfL, UCHL1 and t-tau differ in concentration in FRDA compared to age-matched controls using a novel Single molecule array (Simoa) multiplex immunoassay. Furthermore, the promising NfL results along with the growing body of literature on NfL as a neurodegeneration marker led us to test the marker in both a singleplex and multiplex context showing similar results.

## Materials and Methods

### Study Population

Plasma samples from 33 consecutive patients with genetically confirmed FRDA enrolled in the European Friedreich’s Ataxia Consortium for Translational Studies (EFACTS) natural history study were collected prospectively at the National Hospital for Neurology and Neurosurgery, London, between January and March 2018. The 13 age- and gender-matched controls were selected from the control population. The control subjects had no clinical evidence of neurological disease. Both studies were approved by the local ethics committee (REC Number: 10/H0716/51; REC Number: 17/LO/0381). All participants provided written informed consent in accordance with the Declaration of Helsinki.

Demographical data (age and gender) was documented for patients and controls. The 40-point semi-quantitative Scale for the Assessment and Rating of ataxia (SARA) (Schmitz-Hübsch et al., 2006) was obtained at the day of the sample collection by clinicians experienced in the scoring of this clinical scale. Disease duration, expressed as difference between age at sample collection and age at symptom onset in years, was used as an additional surrogate parameter for disease severity. The presence of clinical signs of neuropathy, namely impaired vibration sense and reduced limb reflexes, were abstracted from the relevant subsections of the Inventory of Non-Ataxia Signs (Jacobi et al., 2013). If either one of these subsections was positive, the patient was recorded to have positive clinical signs of neuropathy. The GAA repeat size was abstracted from the EFACTS database.

### Measurement of Plasma Biomarkers

EDTA plasma samples were collected from unfasted participants, processed within one hour of collection and stored at -80°C. Two microcentrifuge tubes were stored for each participant to avoid multiple freeze-thaw cycles. Plasma NfL, GFAP, UCHL1, and t-tau concentrations were quantified using the Simoa Human Neurology A 4-Plex assay (N4PA) (Quanterix^TM^, Lexington, MA, United States) on a Simoa HD-1 analyzer (Quanterix^TM^, Lexington, MA, United States) according to manufacture guidelines. The lower limits of detection for the target proteins are 0.104 pg/mL, 0.221 pg/mL, 0.024 pg/mL, and 1.74 pg/mL for NfL, GFAP, t-tau, and UCHL1, respectively. Additionally, plasma NfL concentrations were quantified a second time in the patient group with the Simoa Neurofilament-light assay (NFLA) (Quanterix^TM^, Lexington, MA, United States), which has a detection limit of 0.038 pg/mL.

All samples were measured in duplicate by aspirating two aliquots from a single well. The mean concentration of the two measurements was used for the analysis. In four patients, only one measurement could be obtained for each protein of interest. Given the low intra-sample variation between duplicate readings for the remaining samples in NfL, GFAP and t-tau, and the lack of reported problems with the single measurements, these values were included in the analysis. Furthermore, the intra-assay coefficient of variation (CV) was calculated between duplicate readings for each protein. In the N4PA, the mean CVs were NfL: 5.0%, GFAP: 3.3%, t-tau: 9.0%, and UCHL1: 23.0%. Samples with CVs above 20% were excluded from further analysis (tau *n* = 3; UCHL1 *n* = 16). Biomarker quantification could not be performed in one patient sample, possibly due to a high lipid content as suspected in the initial visual inspection. The mean CV in the NFLA was 4.2%, with no sample exceeding the CV threshold.

### Statistical Analysis

Descriptive statistics consisted of mean and standard deviation (SD) for normally distributed continuous variables and median and interquartile range (IQR) for skewed continuous variables. All variables were examined for normality with distributional plots and the Shapiro–Wilk test. As biomarker levels were not normally distributed, natural log-transformation was performed and produced normal distribution of all biomarker values. For the simplicity of notation, the original values were used in the text, figures, and tables. The independent Student *t*-test and the Mann–Whitney *U*-test were used to test for inter-group differences for transformed and raw biomarker levels, respectively. The Pearson’s chi-square test was used to assess between-group differences for categorical variables. The relationship between age and biomarker levels was assessed with the Spearman’s correlation coefficient (*rs*). Associations between disease-related parameters and biomarker levels were computed with multiple linear regressions adjusted for age. Due to the significant number of cases for which a GAA repeat size was not available, the relationship between GAA repeat length and transformed biomarkers were computed separately using Pearson’s partial correlations controlling for age. Patients who were heterozygote for a *FXN* point mutation and a GAA repeat expansion were excluded from the correlation test using GAA repeat length. The agreement between log-NfL levels obtained from the N4PA and NFLA was investigated using the Lin’s concordance coefficient. The agreement between the two measurements was visualized by constructing a Bland–Altman plot. Linear regression was used to identify the presence of a proportional bias. All tests were two-sided with a threshold for statistical significance set at *p* < 0.05. Analysis was first performed including two major outliers and repeated after their exclusion to evaluate their influence. Their exclusion did not affect any statistical significances. The statistical analysis was carried out using SPSS Statistics 25.0 (IBM Corporation, NY, United States) and GraphPad Prism 9.4 (The MathWorks Inc., MA, United States).

## Results

### Comparison of Biomarker Concentrations Between Patients and Controls

A total of 46 subjects consisting of 33 individuals with FRDA and 13 healthy controls were enrolled. An overview of demographic and clinical characteristics of the groups is provided in Tables 1, 2. The study population was predominantly female (59%), with a median age of 35.50 (IQR: 24.75–46.50) years. There was no statistically significant difference in age (*p* = 0.825) or gender (*p* = 0.675) between controls and patients. The median age of onset was 13 (7.50–22.50) years, with six (18%) individuals classifying as late-onset (>25 years) FRDA. The mean total SARA score (±SD) was 20.44 ± 7.40. The SARA score correlated directly with the disease duration (*rs* = 0.57, *p* < 0.001). The genetic dataset in the EFACTS database was available for 24 (72.3%) patients. Two of these patients were compound heterozygote, harboring a point mutation on one *FXN* allele and an expanded GAA repeat on the other. The remaining patients were homozygous for expanded GAA repeats. All patients had clinical signs of neuropathy at the time of sample collection.

**Table 1:** Demographics.

	Controls	FRDA	*P* value
**Gender**			
Male	6 (46.2%)	13 (39.4%)	0.675
Female	7 (53.8%)	20 (60.6%)	
**Age (years)**	37 (29.00–42.50)	34 (23.50–40.00)	0.825


*Data are shown as median (interquartile range) or absolute number (%).*

**Table 2:** Summary of clinical characteristics.

	*N* Patients	Mean ±*SD*	Range
AOO (years)^∗^	33	13 (7.50–22.50)	2–51
Disease duration (years)^∗^	33	18 (12.00–25.00)	6–61
GAA1 size^#^	24	584.40 ± 64.55	150–1200
GAA2 size^#^	24	817.20 ± 54.17	534–1200
SARA score	33	20.44 ± 7.40	6.50–33.00
Neuropathy	33	33 (100%)	–


*‘^∗^’ Median and interquartile range are given for these variables due to skewed distribution. ‘^#^’ Patients who were heterozygote for a *FXN* point mutation and a GAA expansions were excluded from this analysis. AOO, Age of onset. SARA, Scale for the Assessment and rating of ataxia. GAA1, *FXN* allele with the shorter GAA repeat length. GAA2, *FXN* allele with the longer GAA repeat length.*

In order to identify possible confounding effects of demographic factors on biomarker values, the relationship between age and gender with protein levels was computed. There was a significant positive association between GFAP levels and age in the patient group (*rs* = 0.626, *p* < 0.001), but not in the control group (*rs* = 0.12, *p* = 0.712). Conversely, NfL values correlated directly with age in the control group (*rs* = 0.740, *p* = 0.006), but not in the patient group (*rs* = -0.070, *p* = 0.707). Neither UCHL1, nor tau levels showed an association with age in either subject group. There was no difference in biomarker concentrations between male and female participants.

**FIGURE 2:**
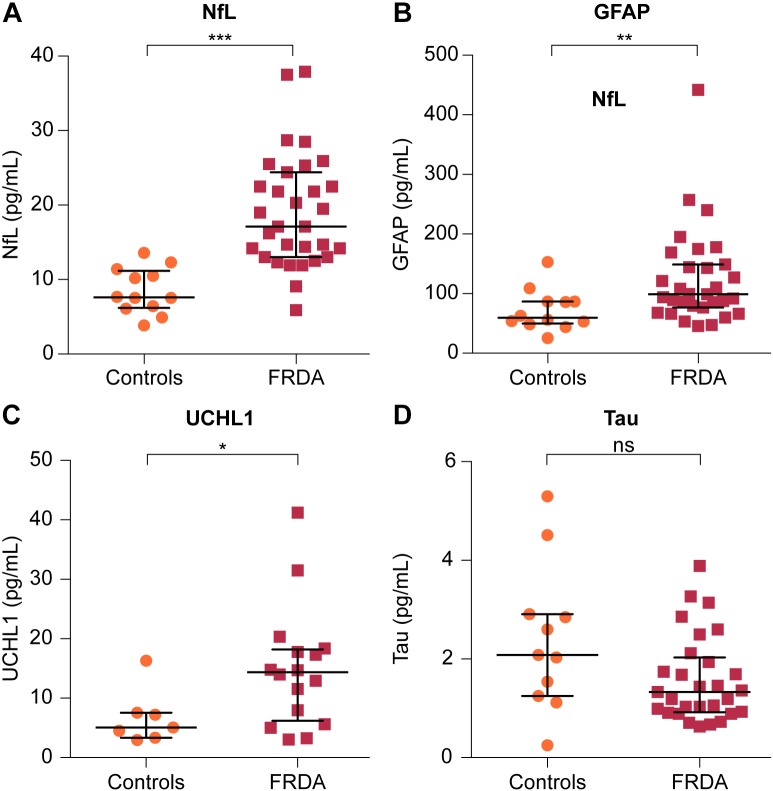
Comparison of plasma levels of four brain-derived proteins between FRDA patients and controls. Plasma concentrations of **(A)** NfL, **(B)** GFAP, and **(C)** ubiquitin C-terminal hydrolase L1 (UCHL1) are significantly increased in FRDA compared to age-matched controls. **(D)** Plasma tau levels were lower in FRDA patients, although this did not reach statistical significance. The horizontal bars indicate the median with error bars denoting the interquartile range. ^∗^*p* < 0.05, ^∗∗^*p* < 0.01, and ^∗∗∗^*p* < 0.001.

Plasma NfL and GFAP concentrations were significantly higher in FRDA patients than in controls (17.10 vs. 7.61 pg/mL, *p* < 0.001 and 99.00 vs. 59.50 pg/mL, *p* = 0.006, respectively). FRDA patients also had significantly higher UCHL1 concentrations compared to the control group (14.79 vs. 5.07 log pg/mL, *p* = 0.020). Conversely, t-tau levels did not differ between patients and controls (1.33 vs. 2.08 pg/mL, *p* = 0.236) (Figure 2 and Table 3).

**Table 3:** Intergroup comparison of plasma biomarker levels.

Biomarker (pg/mL)	Controls	Patients
NfL	7.61 (6.18–11.18)	17.10 (13.00–24.40)
GFAP	59.50 (49.75–86.88)	99.00 (76.50–149.00)
T-tau	2.08 (1.25–2.91)	1.33 (0.92–2.03)
UCHL1	5.07 (3.33–7.55)	14.79 (7.96–31.50)


*Median (interquartile range) biomarker levels of untransformed data in the control and the patient group are outlined. Neurofilament-light (NfL), glial fibrillary acidic protein (GFAP) and ubiquitin C-terminal hydrolase L1 (UCHL1) were significantly raised in FRDA patients compared to controls.*

### Association of Biomarkers With Measures of Disease Severity

Multiple linear regressions adjusted for age were used to model the relationship between the SARA score and the disease duration with biomarker concentrations (outcome). A significant regression equation was found for GFAP concentrations [*F*(3,27) = 11.43, *R*^2^= 0.51, and *p* < 0.001]. The regression equation showed that only age was a significant predictor of GFAP levels (standardized ß-coefficient = 0.60, *p* < 0.003). No other significant associations were found. Plasma concentrations of the four biomarkers did not correlate with the GAA repeat size of either *FXN* allele as assessed by partial rank correlations controlling for age.

### Correlation Between Plasma Markers

**FIGURE 3:**
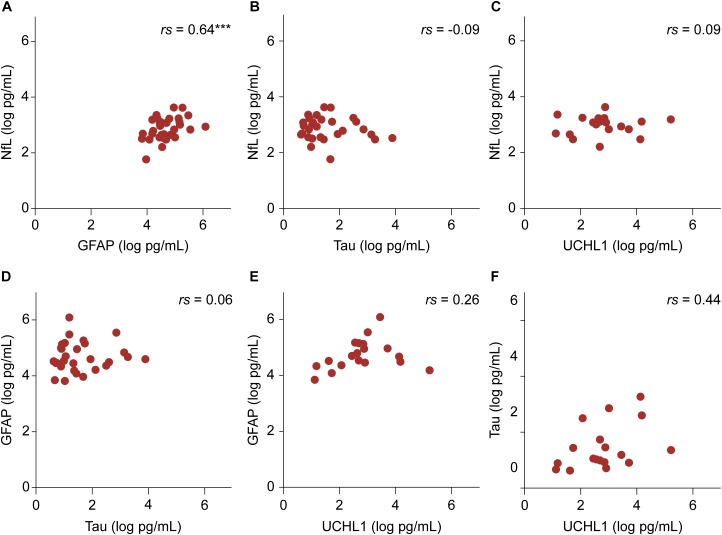
The relationship between transformed biomarker concentrations in the patient group. **(A–F)** Partial correlation controlling for age are shown. The relationship between NfL and GFAP values was significant. ^∗∗∗^*p* < 0.001.

Among the four biomarkers studied, transformed GFAP and NfL values showed a positive linear relationship in the patient group while controlling for age (*r* = 0.64, *p* < 0.001) (Figure 3). In the control group, there were no significant associations among biomarker values.

### Comparison of NfL Values Derived From the N4PA and NFLA

**FIGURE 4:**
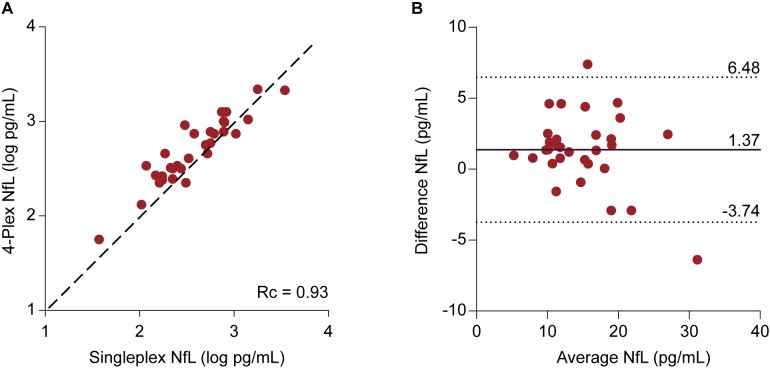
Comparison between NfL measurements derived from the 4-Plex and NfL singleplex assay | **(A)** Individual log**-**transformed NfL from the 4-Plex and NfL singleplex were plotted against each other. Lin’s concordance correlation coefficient (*r_c_*) evaluates the degree to which pairs of measurements fall on the 45° line, here represented as the discontinuous line, through the origin. **(B)** Bland–Altman Plot. Each patient sample is represented as a point on the graph. The difference between the two measurements (Neurology 4-Plex assay – Neurofilament-light singleplex assay) is plotted against the mean of the two measurements [(Neurology 4-Plex assay + NfL singleplex assay)/2]. The mean difference (1.37 pg/mL) is represented by the black line. The upper (6.48 pg/mL) and lower (-3.74 pg/mL) 95% agreement intervals, designated with discontinuous lines, are shown. These statistical limits were computed using the mean and the standard deviation (2.60 pg/mL) of the differences between two measurements.

Samples were included for which NfL values had been obtained from both assays (*n* = 31). The median NfL values were 14.26 (11.31–19.37) pg/mL and 12.43 (9.41–18.71) pg/mL for the N4PA and the NFLA, respectively. The mean difference of 1.37 ± 2.60 pg/mL differed statistically significant from 0 based on a one-sample *t*-test (*p* = 0.006). The Lin’s concordance correlation coefficient showed a very strong concordance between the individual transformed NfL values derived from the two assays (*r_c_* = 0.93, 95% CI: 0.78–0.94) (Figure 4A). A Bland–Altman plot was constructed by plotting the differences against the average NfL values (Figure 4B). The graph indicates that the magnitude of the difference is similar across the range of measured plasma concentrations. This is supported by a linear regression model with the difference of the measurements as dependent and the mean NfL value as independent variable [ß = -0.15, *F*(1,29) = 3.14, and *p* = 0.087].

## Discussion

This study represents the first investigation of plasma markers of neurodegeneration in FRDA. Using the ultrasensitive Simoa technology, we showed that the brain-derived proteins NfL, GFAP, and UCHL1 are significantly raised in FRDA compared to aged-matched controls. Furthermore, it was demonstrated that NfL concentrations derived from the novel N4PA show a strong concordance with those obtained from a well-established NfL singleplex assay. Several conclusions can be drawn from this study, with implications beyond the field of FRDA.

Firstly, our results indicate that the Simoa N4PA has a comparable analytic performance to the Simoa NFLA concerning the measurement of plasma NfL. Previous concerns about multiplex immunoassays evolved mainly around technical challenges, including the interferences of multiple reagents, cross-reactivity between different antibodies and the vast dynamic range of biological proteins (Kingsmore, 2006). To the best of our knowledge, only one study has previously published results using the N4PA in a cohort of 107 adults with traumatic brain injury (Korley et al., 2018). Korley et al. (2018) reported a strong correlation between these two assays with a small mean difference. Indeed, the mean difference of 1.37 pg/mL observed in our patient population is comparable to the 1.87 pg/mL reported in their study. Although Bland–Altman plots are useful in the assessment of agreements between two quantitative measurement methods, it is important to note that the plots do not imply whether the constructed 95% limits of agreements are acceptable in clinical practice (Giavarina, 2015). This range must be defined according to clinical and research necessities in the specific setting. Considering the potential use of NfL to monitor the severity and progression in FRDA, the magnitude of the difference appears to be in a range that is unlikely to be of great significance. However, although our analysis did not reveal the presence of a proportional bias, Korley et al. (2018) reported a greater difference with increasing NfL values, most evidently above 100 pg/mL. The discrepancy between the two studies may be explained by the limited range of NfL values observed in FRDA, which did not exceed 38.7 pg/mL. Therefore a validation of the N4PA assay across a wider concentration range is required.

Secondly, given the complex neuropathology of FRDA and the lack of studies investigating brain-specific proteins as dynamic markers of central nervous system disease progression, the simultaneous measurement of multiple proteins may be beneficial. The N4PA allows the detection of NfL, GFAP, UCHL1, and t-tau in plasma volumes as little as 140 μL in parallel using paramagnetic beads with different coded dye-labels for the four target analytes (Wilson et al., 2016). This yields several advantages, including the need for smaller sample volumes, minimization of processing times, and cost savings. Therefore, it can be hypothesized that the quantification of several proteins in one single experiment could surge the discovery of biofluid markers in FRDA and beyond.

In the present study, the concomitant increase of markers for neuroaxonal damage and astrocytic involvements suggests the presence of non-cell autonomous processes in FRDA. In the last decade research on hereditary neurodegenerative disorders has shifted from a neuron-centric view toward the recognition of destructive mechanisms arising from central nervous system glial cells, which likewise express the respective mutant proteins (Lobsiger and Cleveland, 2007; Phatnani and Maniatis, 2015). Consistent with this hypothesis, a parallel rise of neuronal and glial markers has been reported in other neurodegenerative diseases, such as amyotrophic lateral sclerosis (Benninger et al., 2016). Specifically in FRDA, dysfunction in glial cells, most importantly astrocytes, has recently gained increasing attention. First evidence of glial contribution to the nervous system pathology was provided by a *Drosophila* model which displayed FRDA-like symptoms and a reduced life-span after targeted suppression of FXN in glial cell lines (Navarro et al., 2010). More recently, Loriìa and Diìaz-Nido (2015) demonstrated that *FXN* knock-down in human astrocytes induces mitochondrial dysfunction, enhances cell death and disrupts neuron–glial interactions *in vitro*. This is supported by a time-dependent effect of FXN deficiency on growth and survival of developing cerebellar, but intriguingly not forebrain, astrocytes in a fly and a mouse model (Franco et al., 2017). Thus, elevated plasma GFAP could serve as a useful marker reflecting *in vivo* astrocyte pathology as a contributing factor to cerebellar dysfunction in FRDA.

The presence of raised plasma NfL levels in FRDA indicates white matter involvement, as NfL is primarily a marker of white matter axonal damage (Yuan et al., 2012). This is consistent with neuropathology reports describing degeneration of the white matter tracts in the spinal cord, brainstem and cerebellum, while the cerebral and cerebellar hemispheres remain largely intact (Koeppen and Mazurkiewicz, 2013). Moreover, although structural imaging is normal in the early stages of FRDA (Mascalchi, 2013), recent developments in neuroimaging modalities such as diffusion-weighted imaging have revealed white matter changes in cerebral regions where FXN deficiency causes damage without producing significant atrophy (Pagani et al., 2010; Rizzo et al., 2011). Notably, in contrast to most neurodegenerative diseases in which elevated NfL levels have been described, FRDA patients almost invariably develop a sensory peripheral neuropathy characterized by deficient myelination primarily in large myelinated axons (Morral et al., 2010). Knowing that NfL is also expressed in the peripheral nervous system (Trojanowski et al., 1986), it is not surprising that elevated plasma NfL levels have recently been reported in peripheral neuropathies without central nervous involvement (Bischof et al., 2017; Sandelius et al., 2018). This raises the question whether the increase in plasma NfL in FRDA is predominately caused by the peripheral or the central nervous system involvement. The investigation of a possible concomitant elevation of other neuronal-specific intermediate filament proteins, such as peripherin and α-internexin, might assist in answering this question. Peripherin and α-internexin are considered as the fourth subunits of the neurofilament multimer in the mature nervous system (Zhao and Liem, 2016). As the name suggests, peripherin is predominantly expressed in the peripheral nervous system, while α-internexin is found in the central nervous system. These intermediate filament proteins have not been investigated as biofluid markers yet. In our cohort, all patients displayed clinical signs of neuropathy. This unfortunately meant that a comparison between a group with and without peripheral pathology was not possible.

In addition to elevated levels of plasma NfL, we also observed increased concentrations of UCHL1. UCHL1 is highly specific to neurons (Day and Thompson, 2010), where it is involved in the ubiquitin-proteasome pathway (UPP) that is crucial for the degrading of intracellular proteins, such as FXN (Rufini et al., 2011). Although UCHL1 has been suggested as a nonspecific maker for neurodegeneration, Öhrfelt et al. (2016) have hypothesized that elevated UCHL1 concentrations could also reflect disturbed proteasomal degeneration. Although UCHL1 has not been specifically studied in FRDA, the expression of enzymes involved in the UPP may be altered due to the degradation of FXN. Therefore, a more detailed characterization of proteins involved in FXN degradation might provide answers to the question whether increased UCHL1 levels in FRDA reflect an altered expression of this protein or non-specific neuronal damage. Moreover, biomarkers involved in the UPP could serve especially useful in FRDA considering that several promising targets within the UPP system have recently been explored in preclinical studies (Rufini et al., 2015; Benini et al., 2017).

Interestingly, the elevation of these markers of neuroaxonal damage was not accompanied by a corresponding increase of t-tau. Differences between these markers may indicate distinct predilection for sites and mechanisms of axonal injury in FRDA. While UCHL1 represents one of the most abundant central nervous system proteins (Bishop et al., 2016) and NFL is primarily expressed in large-caliber myelinated axons, t-tau is regarded as a marker for damage to thin, unmyelinated axons (Yilmaz et al., 2017). These unmyelinated axons are not specifically vulnerable in FRDA (Koeppen and Mazurkiewicz, 2013). Therefore, the lack of changes of tau in FRDA patient blood is not surprising. Moreover, clearance mechanism of tau in the peripheral circulation may complicate its interpretation in slowly progressive neurological disease, such as FRDA (Zetterberg et al., 2013). These degrading mechanisms might explain seemingly inconsistent reports regarding the relationship between t-tau levels measured in plasma and in CSF. While good correlations have been described in acute or rapidly progressive neurological diseases (De Vos et al., 2017; Kovacs et al., 2017), weak or absent correlations have been reported in slowly progressive neurodegenerative disorders (Zetterberg et al., 2013; Mattsson et al., 2016).

With the main objective of biomarker research in FRDA being focused on identifying markers of disease progression, we investigated the relationship between clinical markers for disease severity with biomarker concentrations. In our FRDA cohort, no significant relationship was observed between disease duration or the SARA score with biomarker levels after adjusting for age. GFAP values initially appeared to be correlated with longer disease duration. However, when we adjusted the multiple regressions to control for age, this correlation no longer significant. This suggests that in our cohort GFAP levels were associated mainly with age rather than with disease duration. Similar observations have previously been made in a small cohort of spinocerebellar ataxia patients, and it has been suggested that GFAP may not only be marker of astrocytic damage, but instead also a marker of astrocyte activation (Shi et al., 2015). This would explain why concentrations do not change in parallel with the neurodegenerative process. Another possible explanation for the lack of correlation with disease severity markers could be the small sample size and the relatively homogeneity of our patient cohort regarding disease duration, with more than 50% of patients having a disease duration between 10 and 19 years.

Indeed, the two most important limitations of this study are its moderate cohort size, especially the low number of controls, and the limited range of disease severity across the cohort. Even though no significant correlation between clinical measurements of disease severity with biomarker levels was observed in this FRDA population, this could be a result of decreased power secondary to these limitations. Moreover, although the analytic performance of the N4PA in measuring NfL was compared to a well-established singleplex assay, we have not yet compared the measurements of the three other proteins to reference assays. Thus, it is clear that no statements can be made about the analytic sensitivity regarding GFAP, t-tau and UCHL1. It is especially clear that further studies are necessary to evaluate the analytic performance of the 4-plex assay in measuring UCHL1 as this protein displayed a considerable higher mean intra-assay CV of 23%, with 16 out of 46 samples being excluded on the basis of not meeting the predefined CV threshold. This observation mirrors the findings from N4PA measurements in a spinocerebellar ataxia type three cohort at our institute (personal communication P Giunti) and undoubtedly requires further investigation.

## Conclusion

This study provides the first assessment of plasma markers of neurodegeneration in FRDA, illustrating that NfL, GFAP, and UCHL1 are significantly raised in FRDA compared to aged-matched control. These observations may serve as the basis of further exploration of these brain-derived proteins as promising biomarkers in FRDA. In addition, we show for the first time *in vivo* an increase of GFAP reflecting astrocyte activation. This is confirmatory of *in vitro* studies suggesting a role of astrocytes in FRDA pathology. Finally, UCHL1 increase may reflect non-specific neuronal damage or alterations in the UPP. Future studies are needed to confirm our findings and determine whether, when applied to more heterogeneous cohorts, they serve as useful markers of disease severity.

## Author Contributions

AZ performed the experiments, analyzed the data, and wrote the manuscript. HG-M and GT-B contributed to materials and writing of the manuscript. MF and AH advised on experimental design and analysis. HZ provided advice on the experiments and revised the manuscript. PG contributed to the design of the study and revision of the manuscript.

## Conflict of Interest Statement

HZ has served at advisory boards for CogRx, Samumed, Roche Diagnostics, Eli Lilly and Wave, has received travel support from Teva and is a co-founder of Brain Biomarker Solutions in Gothenburg AB, a GU Ventures-based platform company at the University of Gothenburg. The remaining authors declare that the research was conducted in the absence of any commercial or financial relationships that could be construed as a potential conflict of interest.

## Abbreviations

CSF: Cerebrospinal fluid
FRDA: Friedreich’s ataxia
FXN: frataxin
GFAP: glial fibrillary acidic protein
N4PA: Neurology A 4-Plex assay
NfL: Neurofilament-light chain
NFLA: Neurofilament-light singleplex assay
SARA: Scale for the Assessment and Rating of ataxia
Simoa: Single-molecule array
T-tau: total tau
UCHL1: ubiquitin carboxy-terminal hydrolase L1
